# Arson and Schizophrenia: A Case Report and Review of Literature

**DOI:** 10.1192/j.eurpsy.2022.891

**Published:** 2022-09-01

**Authors:** G. Gill, S. Rothman, G. Yadav, P. Riess

**Affiliations:** 1 Bronx Care health System, Psychiatry, Bronx, United States of America; 2 BronxCare Health System, Psychiatry, New York, United States of America

## Abstract

**Introduction:**

Firesetting is a behavior, arson is a crime, and pyromania is a psychiatric diagnosis. Arson is a criminal act in which a person or group of persons willing fully and maliciously sets fire or aid in firesetting to cause harm to property, people, and infrastructure. The likelihood of an arson offender having schizophrenia is 20 times greater than that in the general population. Here, we describe the case of a male in his 50’s, a first-time arsonist, who suffered from schizophrenia since his late teens prior to the onset of random firesetting behavior.

**Objectives:**

To understand the association between Arson and Schizophrenia.

**Methods:**

A case report, as well as a review of the literature, was conducted.

**Results:**

The patient is a male in his 50’s carrying a diagnosis of Schizophrenia with over 50 inpatient hospitalizations. He was observed standing on the threshold of a neighbor’s apartments where he allegedly set fire to a pile of clothing. These charges are based upon allegations that he attempted to set fire to a 14-storey apartment building. At the time of his assessment, he was floridly psychotic. He was found not fit to stand trial. He was restarted on Clozapine and Depakote which is the medication he had the most success with.

**Conclusions:**

Literature shows that Arson and firesetting behaviors are quite commonly seen in patients with mental disorders. Arson often has sequelae that negatively impact the community. The strong correlation between firesetting behavior and mental disorders needs extensive, detailed collaboration between psychiatry, legal expertise, and fire services.

**Disclosure:**

No significant relationships.

